# Isolation of Drug-Resistant *Gallibacterium anatis* from Calves with Unresponsive Bronchopneumonia, Belgium

**DOI:** 10.3201/eid2604.190962

**Published:** 2020-04

**Authors:** Laura Van Driessche, Kevin Vanneste, Bert Bogaerts, Sigrid C.J. De Keersmaecker, Nancy H. Roosens, Freddy Haesebrouck, Lieze De Cremer, Piet Deprez, Bart Pardon, Filip Boyen

**Affiliations:** Author affiliations: Ghent University, Merelbeke, Belgium (L. Van Driessche, F. Haesebrouck, L. De Cremer, P. Deprez, B. Pardon, F. Boyen); Sciensano, Brussels, Belgium (K. Vanneste, B. Bogaerts, S.C.J. De Keersmaecker, N.H. Roosens)

**Keywords:** Gallibacterium anatis, infectious bronchopneumonia, cattle, zoonoses, respiratory diseases, antimicrobial resistance, extensively drug-resistant, multidrug-resistant, whole-genome sequencing, therapy failure, MALDI-TOF, mass spectrometry, Belgium

## Abstract

*Gallibacterium anatis* is an opportunistic pathogen, previously associated with deaths in poultry, domestic birds, and occasionally humans. We obtained *G. anatis* isolates from bronchoalveolar lavage samples of 10 calves with bronchopneumonia unresponsive to antimicrobial therapy. Collected isolates were multidrug-resistant to extensively drug-resistant, exhibiting resistance against 5–7 classes of antimicrobial drugs. Whole-genome sequencing revealed 24 different antimicrobial-resistance determinants, including genes not previously described in the *Gallibacterium* genus or even the *Pasteurellaceae* family, such as *aad*A23, *bla*_CARB-8_, *tet*(Y), and *qnr*D1. Some resistance genes were closely linked in resistance gene cassettes with either transposases in close proximity or situated on putative mobile elements or predicted plasmids. Single-nucleotide polymorphism genotyping revealed large genetic variation between the *G. anatis* isolates, including isolates retrieved from the same farm. *G. anatis* might play a hitherto unrecognized role as a respiratory pathogen and resistance gene reservoir in cattle and has unknown zoonotic potential.

Infectious bronchopneumonia has a major economic impact, causing high morbidity and mortality rates in cattle production systems worldwide (*1*). Furthermore, it is the main indication for antimicrobial use in calves and youngstock (*2*), often resulting in acquired antimicrobial resistance (AMR) among bovine respiratory pathogens (*3*). Bacterial pathogens commonly involved in bronchopneumonia in cattle are *Histophilus somni*, *Mannheimia haemolytica*, *Mycoplasma bovis*, and *Pasteurella multocida* (*4*).

*Gallibacterium anatis*, a gram-negative coccobacillus within the family *Pasteurellaceae*, is historically considered an opportunistic pathogen of intensively reared poultry and domestic birds, where it is mainly isolated from the upper respiratory and lower genital tracts (*5*). *G. anatis* has emerged as a multidrug-resistant pathogen in poultry, mainly causing salpingitis (*6*), resulting in decreased egg production and increased mortality rates (*7*) but also peritonitis (*8*), epididymitis (*6*), and respiratory tract lesions (*9*). In humans, *G. anatis* has been occasionally associated with chronic bronchitis (*10*), lung abscesses (*11*), bacteremia, and death (*12*).

*G. anatis* has rarely been isolated in Belgium, from bovine feces (*13*) or from unknown sources (*13*,*14*), but has not, to the authors’ knowledge, been reported from nasopharyngeal and tracheal bacterial communities of healthy cattle or cattle with bacterial bronchopneumonia (*15*). Therefore, whether *G. anatis* plays a role in the bovine respiratory disease complex as a facultative pathogenic bacterium remains unclear. Our study reports the detection of multiple independent *G. anatis* isolates from cattle with unresponsive infectious bronchopneumonia; our findings are supported by whole-genome sequencing (WGS) to characterize AMR and genetic relatedness.

## Materials and Methods

### Animal Sampling

We retrieved *G. anatis* isolates during a 2-year period (2017–2018) from 10 calves from 7 unrelated farms in Belgium; all 10 calves had a history of respiratory problems (≈5% of the total amount of samples). No poultry was present at these farms; however, at farm 2 (Table 1), raw eggs were occasionally fed to the calves. We obtained all isolates from animals 4–60 days old (Table 1) exhibiting signs of infectious bronchopneumonia, such as fever (>39.3°C), cough, nasal discharge, depression, and adventitious lung sounds. Before the sampling, each calf had already been treated unsuccessfully with first- or second-line antimicrobial drugs. Thoracic ultrasound examination, performed with a 7.5-MHz linear probe as described previously (*16*), showed a consolidated zone in the lung of >1 cm^3^ in all animals. A nonendoscopic bronchoalveolar lavage (nBAL) was conducted in all cases, as described previously (*17*). The sampling method was approved by the ethics committee of the Faculty of Veterinary Medicine, Ghent University (approval no. EC 2016/20).

**Table 1:** Origin and characteristics of *Gallibacterium anatis* strains isolated from calves with unresponsive bronchopneumonia, Belgium, 2017–2018*

Isolate	Age of calf, d	Type (breed)	Farm	Culture	Other pathogens detected	MALDI-TOF MS log score†
GB2	36	Beef (BWB)	1	Pure culture	ND	2.40
GB3	20	Beef (BWB)	2	Dominant isolate	*Escherichia coli*	2.13
GB4	14	Beef (BWB)	2	Pure culture	ND	2.48
GB5	15	Beef (BWB)	2	Pure culture	ND	2.46
GB6	18	Beef (BWB)	2	Dominant isolate	*Histophilus somni*	2.47
GB7	60	Beef (BWB)	3	Dominant isolate	*Bibersteinia trehalosi*, *Mycoplasma bovis*	2.34
GB8	22	Beef (BWB)	4	Dominant isolate	*Trueperella pyogenes*	2.38
GB9	40	Beef (BWB)	5	Pure culture	ND	2.38
GB10	23	Beef (Blonde d’Aquitaine)	6	Dominant isolate	*Mannheimia haemolytica*, *M. bovis*	2.23
GB11	4	Dairy (Holstein Friesian)	7	Pure culture	ND	2.24

*BWB, Belgian White and Blue; MALDI-TOF MS, matrix-assisted laser desorption/ionization time-of-flight mass spectrometry; ND, not detected. †Identification with a log score value >2.0 is considered reliable at the species level.

### Identification

We inoculated all nBAL samples on an Oxoid Columbia blood agar enriched with 5% sheep blood (http://www.oxoid.com) and on a BD Difco modified pleuropneumonia-like organism agar plate (https://www.bd.com) containing 832,000 IU/L polymyxin, 0.36 g/L ampicillin, 23.1% deactivated horse serum, and 6.5% yeast extract for the isolation of *Mycoplasma* spp. We incubated blood agar plates overnight and pleuropneumonia-like organism agars for 5 days, both at 35°C and in a 5% CO_2_ enriched atmosphere. We identified bacterial colonies, grown on both agars, with matrix-assisted laser desorption/ionization time-of-flight mass spectrometry by using the direct transfer method and α‐cyano‐4‐hydroxycinnamic acid as matrix, according to the manufacturer’s guidelines. We considered identifications with a log score value >2.0 to be reliable at the species level. We subcultured *G. anatis* isolates on Columbia blood agar enriched with 5% sheep blood (Oxoid) to obtain a pure culture, which we stored at −80°C for further analysis.

### Antimicrobial-Susceptibility Testing

For susceptibility testing, we performed the broth microdilution technique for ampicillin, ceftiofur, doxycycline, enrofloxacin, florfenicol, gentamicin, kanamycin, penicillin, spectinomycin, tetracycline, tilmicosin, trimethoprim/sulfamethoxazole, tulathromycin, and tylosin, according to Clinical and Laboratory Standards Institute standards (*18*,*19*). Concentrations of all antimicrobial drugs ranged from <0.03 to >128 μg/mL. We performed susceptibility testing of amoxicillin/clavulanic acid by using the gradient strip test. We used *Escherichia coli* ATCC 25922 and *Staphylococcus aureus* ATCC 29213 as quality-control strains. In addition, we included *E. coli* ATCC 35218 as the quality-control strain for amoxicillin/clavulanic acid testing. We used ampicillin, tetracycline, enrofloxacin, tylosin, florfenicol, spectinomycin, and trimethoprim/sulfamethoxazole as class representatives of the penicillins, tetracyclines, fluoroquinolones, macrolides, phenicols, aminocyclitol/aminoglycosides, and potentiated sulphonamides, respectively, to determine phenotypic resistance for these classes, using Clinical and Laboratory Standards Institute breakpoints for *G. anatis* (Appendix Table 1) (*18*).

### Whole-Genome Sequencing

We prepared genomic DNA by using the Bioline Isolate II Genomic DNA kit (Meridian Bioscience, https://www.meridianbioscience.com), following the manufacturer’s instructions. We constructed sequencing libraries by using the Illumina Nextera XT DNA sample preparation kit and then sequenced isolates using the MiSeq Reagent v3 kit with a 250-bp paired-end protocol (Illumina, https://www.illumina.com) according to the manufacturer’s instructions. We have deposited all generated WGS data in the National Center for Biotechnology Information Sequence Read Archive (*20*) under accession number PRJNA541488. We cleaned and assembled raw reads (Appendix Table 2) and used Kraken 0.10.5 (*21*) to perform k-mer–based classification of cleaned reads against an in-house dump of the complete genomes from the National Center for Biotechnology Information RefSeq Microbial Genomes Database (*22*). We analyzed paired-end reads and orphaned reads (i.e., reads where only 1 read of the pair survived cleaning) separately by using default settings and then combining the results by concatenating the output files.

### Antimicrobial-Resistance Genotyping

We performed genotypic resistance gene detection, as described by Bogaerts et al. (*23*), against the ResFinder database (*24*). We defined AMR gene clusters as resistance genes on the same contig within a sample. We performed detection of mutations linked with increased fluoroquinolone MICs in the quinolone-resistance determining regions of *gyrA* and *parC* by aligning these regions in the *E. coli* K12 reference genome in NCBI (accession no. NC_000913.3) for *gyrA* (accession no. NP_416734) and *parC* (accession no. NP_417491.1) by using the Needle tool for pairwise sequence alignment of the EMBOSS suite (https://www.ebi.ac.uk/tools/psa) (*25*). We used mlplasmids 1.0.0 (https://sarredondo.shinyapps.io/mlplasmids) to predict whether assembled contigs were either plasmid- or chromosome-derived, by using *E. coli* as species model and 1,000 bp as the minimum sequence length (*26*). We then compared contigs predicted to be plasmid-encoded by using blastn (https://blast.ncbi.nlm.nih.gov/Blast.cgi), with default settings, against the nucleotide database. We performed transposase detection by using ISFinder (https://www-is.biotoul.fr/index.php) with the blastn tool, using default settings (*27*), to substantiate the presence of transposable elements in close proximity to the AMR gene clusters in the specific contigs of the whole assembly. Last, we used ICEberg 2.0 (http://db-mml.sjtu.edu.cn/ICEberg), with default settings, to detect integrative and conjugative elements (ICEs) or integrative and mobilizable elements (IMEs) in the *G. anatis* assemblies (*28*).

### Sample Relatedness

For multilocus sequence typing (MLST), we used an in-house copy of the MLST database for *G. anatis* hosted by the PubMLST platform (http://pubMLST.org/anatis) (*29*), which we pulled in-house using the REST API (*30*), for MLST genotyping. We typed individual loci separately by aligning the assembly for each sample against all allele sequences of that locus by using nucleotide BLAST+ 2.6.0, with default values (*31*). We then performed filtering and best hit identification, as described previously, for AMR gene characterization. Because MLST offered limited resolution in the relationship between samples, we used a single-nucleotide polymorphism (SNP) genotyping approach based on an in-house implementation of the CSI Phylogeny workflow (https://omictools.com/csi-phylogeny-tool) (Appendix Table 3) (*32*), using the NCBI RefSeq entry for *G. anatis* (accession no. NC_015460) as reference to compare diversity among samples. We used MEGA-Computing Core 10.0.4 (https://www.megasoftware.net) to detect the best evolutionary model and construct a maximum-likelihood phylogenetic tree on the basis of the SNP matrix, setting the following options: “missing-data” set to “partial_deletion,” “site-cov-cutoff” set to 50, “branch-swap” set to “very_weak,” “ml-method” set to “spr3,” “action” set to “model,” and “bootstraps” set to 100. We then repeated the same workflow by using the genome assembly of isolate GB8 (Appendix Table 3), filtered on contigs >1,000 bases with a k-mer coverage of 10–50× as reference. We visualized the resulting phylogenetic trees by using iTOL (*33*) and, afterward, a midpoint rooting. In addition, we constructed a core genome MLST (cgMLST) scheme to investigate the relationship of the isolates in Belgium compared with all genomes for this species publicly available in the NCBI database (Appendix Table 4).

## Results

### Identification

We compiled all strain origin information and co-infection data (Table 1). The *G. anatis* isolates were all nonhemolytic and were recovered as a pure culture (50% of cases) or the predominant isolate in large numbers (50% of cases). When a dominant culture was obtained, other pathogens were detected to a lesser extent. All calves recovered from the pneumonia because of appropriate antimicrobial therapy, except 1 who was euthanized because of cardiac failure.

### Antimicrobial Susceptibility Testing

We observed high MIC values for tylosin, tetracycline, spectinomycin, kanamycin, and enrofloxacin for all isolates, which most likely explains therapeutic failure (Table 2; Appendix Table 1). All isolates exhibited very low MIC values for ceftiofur and amoxicillin/clavulanic acid.

**Table 2:** Overview of phenotypic and genotypic resistance determinants of all investigated bovine *Gallibacterium anatis* isolates, Belgium, 2017–2018*

Isolate	Antimicrobial classes with phenotypic resistance	Identified genotypic resistance determinants
GB2	Macrolides, potentiated sulphonamides, tetracyclines, phenicols, aminoglycosides, fluoroquinolones	*ermB*, *sul2*, *tetM*, *catA1*, *catA3*, *floR*, *aadA1*, *aadB*, *aphA1*, *strA*, *strB*, *gyrA* 83S→Y, *gyrA* 87D→A, *parC* 80S→I
GB3	Penicillins, macrolides, tetracyclines, phenicols, aminoglycosides, fluoroquinolones	*bla*_CARB-8_, *bla*_TEM-2_, *ermB*, *sul1*, *sul2*, *tetB*, *tetM*, *tetY*, *floR*, *aadA1*, *aadB*, *aphA1*, *strA*, *strB*, *gyrA* 83S→Y, *gyrA* 87D→A, *parC* 80S→I
GB4	Penicillins, macrolides, potentiated sulphonamides, tetracyclines, phenicols, aminoglycosides, fluoroquinolones	*bla*_TEM-2_, *ermB*, *dfrA1*, *sul2*, *tetB*, *tetM*,*catA1*, *aac(6”)-aph(2”)-1*, *aadA1*, *aph(3′)-III*, *strA*, *gyrA* 83S→Y, *gyrA* 87D→A, *parC* 80S→I
GB5	Macrolides, potentiated sulphonamides, tetracyclines, aminoglycosides, fluoroquinolones	*ermB*, *dfrA1*, *sul2*, *tetB*, *tetM*, *catA1*, *floR*, *aadA1*, *aadB*, *aphA1*, *strA*, *gyrA* 83S→Y, *gyrA* 87D→A, *parC* 80S→I
GB6	Penicillins, macrolides, potentiated sulphonamides, tetracyclines, phenicols, aminoglycosides, fluoroquinolones	*bla*_CARB-8_, *bla*_TEM-2_, *ermB*, *dfrA1*, *sul1*, *sul2*, *tetB*, *tetM*, *tetY*, *floR*, *aadA1*, *aphA1*, *strA*, *strB*, *gyrA* 83S→F, *gyrA* 87D→G, *parC* 80S→I
GB7	Penicillins, macrolides, potentiated sulphonamides, tetracyclines, aminoglycosides, fluoroquinolones	*bla*_TEM-2_, *ermB*, *sul2*, *tetB*, *tetM*, *catA1*, *catA3*, *aadA1*, *aadB*, *aphA1*, *strA*, *strB*, *gyrA* 83S→F, *gyrA* 87D→G, *parC* 80S→I
GB8	Penicillins, macrolides, potentiated sulphonamides, tetracyclines, aminoglycosides, fluoroquinolones	*bla*_TEM-2_, *ermB*, *mphE*, *mrsE*, *dfrA1*, *sul2*, *tetB*, t*etM*, *catA1*, *catA3*, *aadA23*, *aadB*,*aphA1*, *strA*, *gyrA* 83S→F, *gyrA* 87D→A, *parC* 80S→I
GB9	Penicillins, macrolides, potentiated sulphonamides, tetracyclines, aminoglycosides, fluoroquinolones	*bla*_TEM-2_, *ermB*, *dfrA1*, *sul2*, *tetB*, *tetM*, *catA1*, *aac(6′)-aph(2”)-1*, *aadA1*, *aph(3′)-III*, *strA*, *gyrA* 83S→Y, *gyrA* 87D→A, *parC* 80S→I
GB10	Penicillins, macrolides, potentiated sulphonamides, tetracyclines, phenicols, aminoglycosides, fluoroquinolones	*ermB*, *sul2*, *tetB*, *tetM*, *catA1*, *floR*, *aadA1*, *aadB*, *aphA1*, *strA*, *qnrD1*, *gyrA* 83S→Y, *gyrA* 87D→A, *parC* 80S→I
GB11	Penicillins, macrolides, potentiated sulphonamides, tetracyclines, aminoglycosides, fluoroquinolones	*bla*_TEM-2_, *ermB*, *dfrA1*, *sul2*, *tetB*, *tetM*, *catA1*, *aac(6′)-aph(2”)*, *aadA1*, *aph(3′)-III*, *strA*, *gyrA* 83S→Y, *gyrA* 87D→A, *parC* 80S→I

*Current Clinical and Laboratory Standards Institute breakpoints for *G. anatis* were used to define susceptibility. Identified resistance genes are listed with their name as present in the ResFinder database. For *gyrA* and *parC*, the resulting amino acid changes at positions 83 and 87 (*gyrA*) and 80 (*parC*) are also indicated.

### Whole-Genome Sequencing

The number of raw paired-end reads, genome assembly length, N50 (a metric used as a proxy for assembly quality that was defined as the length at which contigs of equal or longer length contained >50% of the assembled sequence), and number of contigs >1,000 bases was in the same range for all samples, with a median of 372,623 raw paired-end reads, median assembly length of 2,483,037 bases, median N50 value of 105,124 bases, and median of 58 contigs >1,000 bases across all samples (Appendix Table 2). Genome assembly sizes were close to the expected size of ≈2.69 Mb (*34*), indicating high quality of the WGS run. K-mer–based classification of read content for all isolates confirmed the samples to be *G. anatis*, given that this was the only species identified in the sample having a 5% read cutoff.

### AMR Genotyping

By using the ResFinder database, we detected various AMR determinants in the WGS data for all isolates (Table 2). In total, we detected 24 different resistance genes across all 10 isolates, and several genes were present in multiple isolates. We found all isolates harbored resistance genes targeting aminoglycosides, phenicols, macrolides, sulphonamides, and tetracyclines. Seven isolates also harbored resistance genes such as *bla*_CARB-8_ or *bla*_TEM-2_ targeting β-lactamase–susceptible penicillins. Six isolates contained *dfr*A1, conferring resistance against trimethoprim. Isolate GB10 carried *qnr*D1, a plasmid-mediated quinolone resistance determinant. We found mutations linked with increased fluoroquinolone MICs in the quinolone resistance determining region of *gyrA* and *parC* (*35*) in all isolates, including a single-point mutation in *parC* (Ser-80 to Ile) and 2 mutations in *gyrA* resulting in S-83 to Y or F, and D-87 to A or G, changes. We determined the genotype to phenotype correspondence to be 90% (phenotypic observations might be explained by genotypic detection of corresponding resistance genes). In GB10, we found very high MIC values for penicillin/ampicillin and no corresponding resistance gene. We did find resistance genes without corresponding high MIC values for potentiated sulphonamides in isolate GB3 and for phenicols in isolates GB5, GB7, GB8, GB9, and GB11.

Some resistance genes were closely linked into resistance gene cassettes (Table 3). Overall, we observed a high diversity of resistance genes, both in determinants present in resistance gene clusters and in separate contigs. We detected gene clusters with 3–4 of the same resistance genes found in GB4, GB9, and GB11, and 2 identical resistance genes in GB3 and GB6 (Table 3). In 19 of 20 clusters, we observed a link with transposases in close proximity or localization on putative predicted IMEs, plasmids, or both (Table 3). In addition, we detected a type 4 secretion system not associated with a resistance gene cluster in GB2, GB5, GB7, and GB10 (data not shown).

**Table 3:** Overview of clustered AMR genes in bovine *Gallibacterium anatis* isolates, Belgium, 2017–2018*

Isolate(s)	Clustered AMR genes†	Linked transposases or IME‡	Predicted contig origin§
GB4, GB9, GB11	*aac6-aph2*, *aph3-III*, *ermB*	Putative IME	Chromosome (0.968–0.971)
GB7	*aadA1*, *aadB*, *catA1*	TnAs3 transposase *A. salmonicida*	Chromosome (0.988)
GB2	*aadA1*, *aadB*, *catA1*, *ermB*, *tetM*	TnAs3 transposase *A. salmonicida*	Chromosome (0.965)
GB3	*aadA1*, *aadB*, *sul1*, *tetM*	TnAs3 transposase *A. salmonicida*	Chromosome (0.98)
GB5	*aadA1*, *catA1*, *dfrA1*, *ermB*, *tetM*	TnAs3 transposase *A. salmonicida*	Chromosome (0.979)
GB4, GB9, GB11	*aadA1*, *catA1*, *dfrA1*, *tetM*	TnAs3 transposase *A. salmonicida*	Chromosome (0.99)
GB10	*aadA1*, *catA1*, *ermB*, *tetM*	TnAs3 transposase *A. salmonicida*	Chromosome (0.977)
GB6	*aadA1*, *dfrA1*, *ermB*, *floR*, *sul1*, *tetM*	TnAs3 transposase *A. salmonicida*	Chromosome (0.986)
GB8	*aadA23*, *catA1*, *dfrA1*, *ermB*, *tetM*	TnAs3 transposase *A. salmonicida*	Chromosome (0.957)
GB8	*aadB*, *aphA1*	Truncated IS6 family transposase	Chromosome (0.848)
GB5	*aadB*, *floR*	IS6 family transposase	Plasmid (0.694); *B. trehalosi* pCCK13698 (75%–99%)
GB7	*aphA1*, *catA3*, *strA*, *strB*, *sul2*	ISapl1 transposase *A. pleuropneumoniae*	Chromosome (0.988)
GB2	*aphA1*, *catA3*, *strA*, *strB*, *sul2*	Truncated IS4 family transposase	Plasmid (0.749); uncultured *Eubacterium* pIE1130 (84%, 99%)
GB10	*aphA1*, *floR*, *strA*, *tetB*	ISVsa3 transposase *V. salmonicida*	Plasmid (0.807); *B. trehalosi* USDA-ARS-USMARC-192 (68%, 99%)
GB3, GB6	*aphA1*, *sul2*	Truncated ISVsa3 transposase *V. salmonicida*	Plasmid (0.898); *P. multocida* USDA-ARS-USMARC-60675 (83%, 99%)
GB4, GB9, GB11	*bla*_TEM-2_, *strA*, *sul2*, *tetB*	Tn3 transposase *Salmonella*	Plasmid (0.864–0.895); *S. sonnei* p866 (83%, 99%)
GB3, GB6	*bla*_TEM-2_, *tetB*	Tn3 transposase *Salmonella*	Chromosome (0.976)
GB7	*bla*_TEM-2_, *tetB*	Tn3 transposase *Salmonella*	Plasmid (0.708); *Salmonella* Heidelberg pN13–01290_23 (100%, 99%)
GB8	*catA3*, *mphE*, *msrE*, *strA*, *sul2*, *tetB*	Truncated ISVsa5 transposase *V. salmonicida*	Plasmid (0.738); *P. multocida* 14424 (71%, 99%)
GB5	*strA*, *tetB*	Not detected	Chromosome (0.526)

*Includes predicted transposases in close proximity of the resistance gene clusters (or predicted IME containing the AMR gene cluster) and the predicated contig origin. AMR, antimicrobial resistance; IME, integrative mobilizable elements. †AMR genes present on the same contig (genes are listed in alphabetical order). ‡Determined by using ISfinder for transposases and ICEberg for IME. §Determined by using mlplasmids. Values in parentheses indicate (range of) posterior probability of belonging to either a plasmid or chromosome. For predicted plasmids, the best hit in the National Center for Biotechnology Information nucleotide database is also listed, with its corresponding query coverage and percentage identity, respectively, in parentheses.

### Sample Relatedness

To evaluate the relationship between isolates, we performed MLST by using the public *G. anatis* database hosted by the PubMLST platform. However, an exact allelic match could only be identified for 1 locus in GB2, 2 loci in GB3, 2 loci in GB4, 3 loci in GB 5, 2 loci in GB6, 1 locus in GB7, 1 locus in GB8, 2 loci in GB9, 1 locus in GB10, and 2 loci in GB11 (in a total of 8 loci in the scheme). Reliable allele calling for the remaining loci was not possible because of mismatches and different lengths for all samples. Closer inspection revealed that the MLST database only contained 89 isolates corresponding with 81 profiles, suggesting that MLST failed because of the lack of an available background to compare against.

Because MLST was not appropriate for delineating relationships, we performed SNP genotyping by using the NCBI RefSeq reference for *G. anatis* (UMN179). We found 14,583–15,234 SNPs for all samples (Appendix Table 3), resulting in a total SNP matrix of 32,104 positions, indicating large diversity between samples. We repeated the workflow by using the assembly of GB8 (which had the highest original read mapping rate) as a reference; this step ensured that the number of SNPs was not erroneously inflated by taking a reference not suited for SNP genotyping (i.e., a reference too divergent from the actual samples). We found 8,978–11,137 SNPs for all samples (Appendix Table 3), resulting in a total SNP matrix of 25,166 positions, confirming the large genetic diversity among samples. Afterward, we performed model selection and phylogenetic tree reconstruction with MEGA, identifying the general time reversible model as the best fit for both references. 

**Figure 1:**
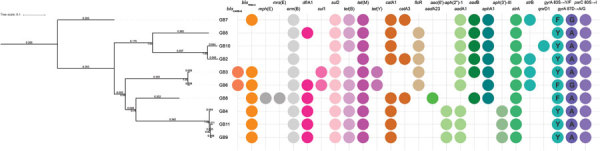
Phylogeny of *Gallibacterium anatis* isolates from cattle in Belgium, 2017–2018, based on single-nucleotide polymorphism genotyping when using GB8 as a reference. Node labels indicate bootstrap support values (expressed as decimals). Branch lengths and the scale bar are expressed as average substitutions per site. The resistance genes detected in each sample are listed to the right according to the legend displayed on top.

We used GB8 as reference for 1 phylogentic tree (Figure 1) and *G. anatis* UMN179 as reference for another (Appendix Figure). Although branch lengths differed, their underlying topology was identical and well supported by high bootstrap values, indicating that, although some isolates clustered together with fewer differences (GB10 with GB2, GB4 with GB9 and GB11, GB3 with GB6), overall we observed large variation between the different isolates. Notably, for the 4 isolates GB3, GB4, GB5, and GB6 obtained from the same farm (Table 2), only GB3 and GB6 clustered together, whereas GB4 and GB5 were located elsewhere in the phylogeny.

**Figure 2:**
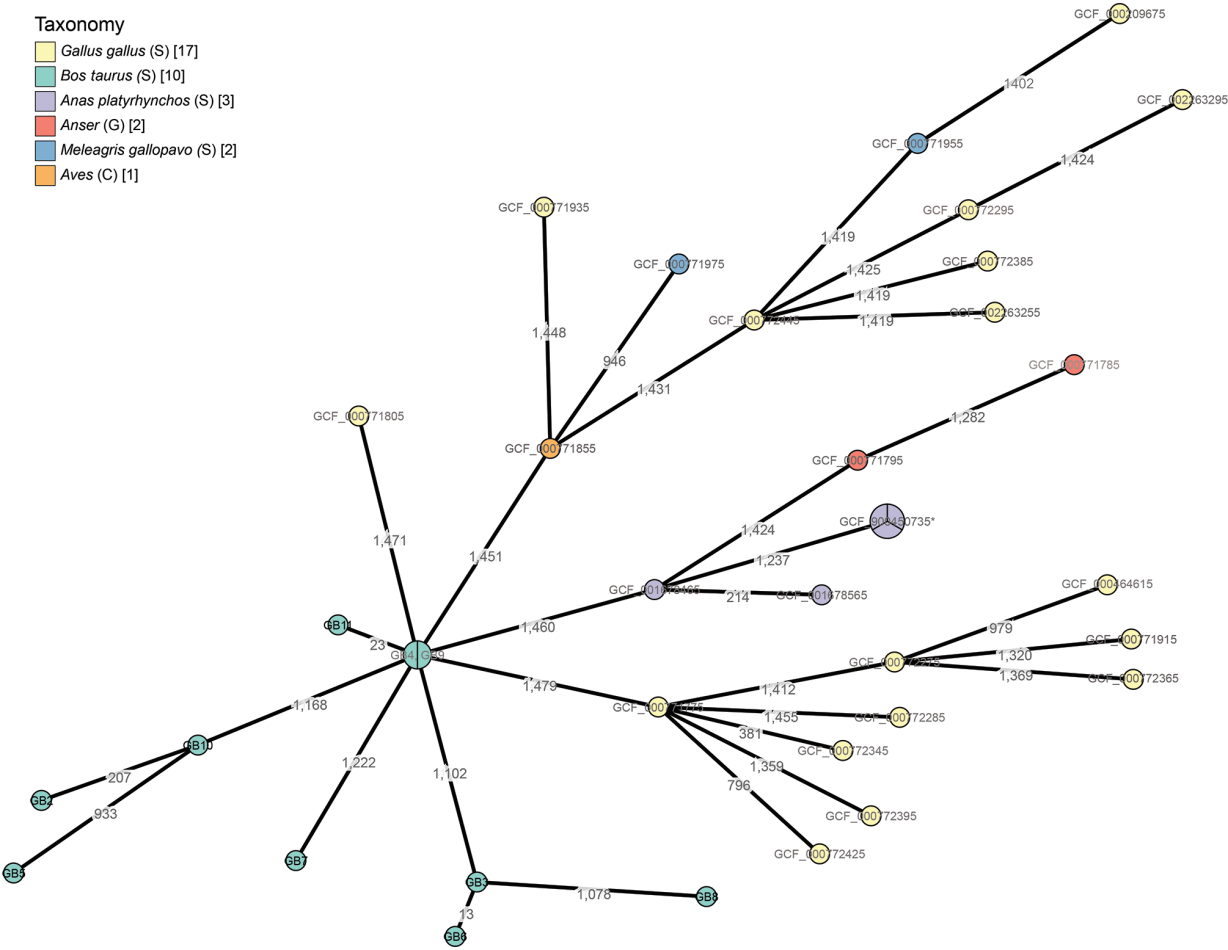
Phylogeny of *Gallibacterium anatis* isolates from cattle in Belgium, 2017–2018, based on a core genome multilocus sequence typing scheme constructed by using the 10 cattle isolates and 27 poultry isolates from National Center for Biotechnology Information (1,516 loci in total). Branch lengths are scaled logarithmically, and branch labels express number of allelic differences between isolates. Nodes scale with the number of isolates that have the same core genome multilocus sequence type. Nodes are colored according to the host organism of the isolate. Asterisk indicates node containing samples GCF_000379785, GCF_000772265, and GCF_900450735 (GB3, GB6, GB8) with the same sequence type. C; class; G, genus; S, species.

We also constructed a cgMLST scheme on the basis of our mining all publicly available *G. anatis* genomes from NCBI, including in total 27 isolates from poultry, complemented with the strains from Belgium (Figure 2). Despite the existence of generally very large distances between all samples, the resulting topology indicated that the strains isolated from cattle in Belgium clustered together and were distinctly separated from all other strains isolated from poultry. Moreover, the subtopology of the isolates from Belgium was concordant with results from the SNP analysis.

## Discussion

Our report illustrates the involvement of *G. anatis* in respiratory disease in cattle. Interestingly, isolation of *G. anatis* from cattle was only described for feces (*13*) or was of unknown origin (*13*,*14*). Also, recent microbiome studies on the nasopharyngeal and tracheal bacterial communities of feedlot cattle did not document the presence of *G. anatis* (*15*). The presence of the bacterium in cattle might have been underestimated in the past, and availability of matrix-assisted laser desorption/ionization time-of-flight mass spectrometry might have improved detection rates for *G. anatis*, as seen in poultry (*36*) and humans (*11*). Nevertheless, finding this bacterium in pneumonic animals on multiple farms suggests the possible emerging nature of this pathogen, as suggested in poultry (*37*). 

In poultry, clonal outbreaks of *G. anatis* have been described (*38*,*39*), in contrast with our study, where both SNP- and cgMLST-based phylogenetic analysis of the cattle isolates demonstrated a high variety between isolates, even for those retrieved on the same farm. This finding indicates that *G. anatis* strains from the different farms do not originate from 1 single introduction or outbreak and that a large unsampled reservoir of circulating *G. anatis* strains exists in cattle within Belgium. Another explanation for retrieving *G. anatis* in calves with pneumonia might be a direct link with poultry on the affected farms. In our study, no poultry was present, nor was poultry manure used as cattle feed at any farm, although at farm 2 (Table 1), raw eggs were occasionally fed to the calves. Because this practice occurred at only 1 farm, an indirect link with poultry seems unlikely. Moreover, cgMLST analysis indicated that, despite the large variation present in the cattle isolates in Belgium, these isolates still clustered together and were clearly separated from all poultry isolates for which genome information was publicly available. The relatively limited number of currently available *G. anatis* genomes and their large overall distances prevent definitive conclusions, but nevertheless support that no direct or indirect link with poultry exists. 

Like other *Pasteurellaceae* species, *G. anatis* most likely acts as an opportunistic bacterium, infecting an already damaged respiratory tract caused by co-infections with viruses or bacteria, as observed in poultry (*37*). Unfortunately, viral involvement in the reported outbreaks in our study cannot be confirmed because we did not perform any viral diagnostics. However, the combined observations we have made suggest that *G. anatis* can act as an opportunistic bacterium in a multifactorial disease complex rather than being a highly virulent pathogen that spreads clonally during a clinical outbreak. To what extent *G. anatis* isolated from cattle in our study can survive in the environment remains unknown.

A second major finding of our study is the multiresistant nature of the retrieved *G. anatis* isolates. All isolates obtained in the study demonstrated acquired resistance against 5–7 different antimicrobial classes, defining them as multidrug-resistant. Although the lack of species-specific clinical breakpoints precludes drawing firm conclusions, the clinical observation of unresponsiveness to antimicrobial treatment with various agents also supports this theory. Because antimicrobial susceptibility testing indicated susceptibility for only cephalosporins, amoxicillin/clavulanic acid, or both in all isolates, the isolates can even be defined as extensively drug-resistant (*40*). Also, for *G. anatis* isolated from poultry, a high prevalence of multidrug resistance has been demonstrated (*37*). However, the isolates retrieved in our study also demonstrated acquired resistance against fluoroquinolones, ampicillin, trimethoprim/sulfamethoxazole, florfenicol, and gentamicin. Furthermore, the level and prevalence of multidrug resistance observed in the *G. anatis* isolates we analyzed surpasses previously described multidrug resistance in bovine *Pasteurellaceae* (*41*–*43*).

We detected >20 different resistance genes in the genomes of the *G. anatis* isolates in our study, including determinants conferring resistance to aminoglycosides, phenicols, macrolides, sulphonamides, trimethoprim, tetracyclines, penicillins, and quinolones. Although many of these resistance genes have been described previously in *Pasteurellaceae* obtained from either animals or humans (*43*,*44*), we detected various other resistance genes not previously reported in *G. anatis* or bovine *Pasteurellaceae*. Moreover, 4 resistance genes have so far never been described in *Pasteurellaceae* at all, namely *aad*A23, *bla*_CARB-8_, *tet*(Y) and *qnr*D1.

In contrast to recently described bovine multidrug-resistant *Pasteurellaceae* (*43*,*45*,*46*), resistance genes in the *G. anatis* isolates in our study were detected at various locations in the genome and were seldom contained within ICE, as described previously for *G. anatis* in poultry (*47*). Only 1 gene cluster, carrying 1 or 2 *erm*(B) copies, as well as *aac*6*-aph*2 and *aph3-III* detected in 3 isolates (GB4, GB9, and GB11), was associated with a predicted putative IME. This putative element did not show any remarkable similarities with any of the IMEs in the ICEfinder database for gram-negative bacteria but did show some similarity with ICEs in *Streptococcus pneumoniae* (data not shown). However, for all remaining clustered resistance genes, we observed a link with transposases, some of which were located on predicted plasmids. In addition, the high prevalence and diversity of resistance genes in the bovine *G. anatis* isolates we analyzed suggests that this species might acquire resistance genes relatively easily compared with other *Pasteurellaceae* species. Indeed, *G. anatis* is considered a naturally competent species that has been demonstrated to be less selective in the uptake of foreign DNA compared with other *Pasteurellaceae* species (*48*). As a consequence, these resistance genes might spread to more pathogenic closely related respiratory bacteria like *Mannheimia haemolytica*, *Histophilus somni*, and *Pasteurella multocida*, possibly leading to therapy failure of infectious bronchopneumonia in cattle. We found no relevant virulence genes in the genomes of the strains in Belgium (Appendix Table 5), indicating that such genes are not present or, alternatively, have not yet been described.

In conclusion, *G. anatis* needs to be taken into account as a secondary respiratory pathogen and resistance gene reservoir in cattle. In addition to poultry, cattle hold a potential risk for zoonotic transmission of *G. anatis*, but further research is required to establish zoonotic potential.

Appendix Additional information about isolation of drug-resistant *Gallibacterium anatis* from calves with unresponsive bronchopneumonia, Belgium.

## References

[1] Snowder GD, Van Vleck LD, Cundiff LV, Bennett GL. Bovine respiratory disease in feedlot cattle: environmental, genetic, and economic factors. J Anim Sci. 2006;84:1999–2008. 10.2527/jas.2006-046 16864858

[2] Pardon B, Catry B, Dewulf J, Persoons D, Hostens M, De Bleecker K, et al. Prospective study on quantitative and qualitative antimicrobial and anti-inflammatory drug use in white veal calves. J Antimicrob Chemother. 2012;67:1027–38. 10.1093/jac/dkr570 22262796

[3] Kehrenberg C, Walker RD, Wu CC, Schwarz S. Antimicrobial resistance in members of the family *Pasteurellaceae*. In: Aarestrup FM, editor. Antimicrobial resistance in bacteria of animal origin. Washington (DC): ASM Press; 2006. p. 167–86.

[4] Griffin D. Bovine pasteurellosis and other bacterial infections of the respiratory tract. Vet Clin North Am Food Anim Pract. 2010;26:57–71. 10.1016/j.cvfa.2009.10.010 20117542

[5] Bisgaard M. Incidence of *Pasteurella haemolytica* in the respiratory tract of apparently healthy chickens and chickens with infectious bronchitis. characterisation of 213 strains. Avian Pathol. 1977;6:285–92. 10.1080/03079457708418238 18770338

[6] Paudel S, Liebhart D, Hess M, Hess C. Pathogenesis of *Gallibacterium anatis* in a natural infection model fulfils Koch’s postulates: 1. Folliculitis and drop in egg production are the predominant effects in specific pathogen free layers. Avian Pathol. 2014;43:443–9. 10.1080/03079457.2014.955782 25144260

[7] Persson G, Bojesen AM. Bacterial determinants of importance in the virulence of *Gallibacterium anatis* in poultry. Vet Res (Faisalabad). 2015;46:57–68. 10.1186/s13567-015-0206-z 26063044 PMC4462078

[8] Jordan FT, Williams NJ, Wattret A, Jones T. Observations on salpingitis, peritonitis and salpingoperitonitis in a layer breeder flock. Vet Rec. 2005;157:573–7. 10.1136/vr.157.19.573 16272543

[9] Mushin R, Weisman Y, Singer N. *Pasteurella haemolytica* found in the respiratory tract of fowl. Avian Dis. 1980;24:162–8. 10.2307/1589775

[10] Gautier AL, Dubois D, Escande F, Avril JL, Trieu-Cuot P, Gaillot O. Rapid and accurate identification of human isolates of *Pasteurella* and related species by sequencing the sodA gene. J Clin Microbiol. 2005;43:2307–14. 10.1128/JCM.43.5.2307-2314.2005 15872260 PMC1153776

[11] de Moreuil C, Héry-Arnaud G, Fangous MS, Le Berre R. [Gallibacterium anatis pulmonary abscess] [in French]. Med Mal Infect. 2017;47:74–6. 10.1016/j.medmal.2016.10.007 27894516

[12] Aubin GG, Haloun A, Treilhaud M, Reynaud A, Corvec S. *Gallibacterium anatis* bacteremia in a human. J Clin Microbiol. 2013;51:3897–9. 10.1128/JCM.01638-13 23966514 PMC3889794

[13] Wang C, Robles F, Ramirez S, Riber AB, Bojesen AM. Culture-independent identification and quantification of *Gallibacterium anatis* (*G. anatis*) by real-time quantitative PCR. Avian Pathol. 2016;45:538–44. 10.1080/03079457.2016.1184743 27171757

[14] Christensen H, Bisgaard M, Bojesen AM, Mutters R, Olsen JE. Genetic relationships among avian isolates classified as *Pasteurella haemolytica*, ‘*Actinobacillus salpingitidis*’ or *Pasteurella anatis* with proposal of *Gallibacterium anatis* gen. nov., comb. nov. and description of additional genomospecies within *Gallibacterium* gen. nov. Int J Syst Evol Microbiol. 2003;53:275–87. 10.1099/ijs.0.02330-0 12656185

[15] Timsit E, Workentine M, van der Meer F, Alexander T. Distinct bacterial metacommunities inhabit the upper and lower respiratory tracts of healthy feedlot cattle and those diagnosed with bronchopneumonia. Vet Microbiol. 2018;221:105–13. 10.1016/j.vetmic.2018.06.007 29981695

[16] Buczinski S, Forté G, Francoz D, Bélanger AM. Comparison of thoracic auscultation, clinical score, and ultrasonography as indicators of bovine respiratory disease in preweaned dairy calves. J Vet Intern Med. 2014;28:234–42. 10.1111/jvim.12251 24236441 PMC4895545

[17] Van Driessche L, Valgaeren B, Schutter PD, Gille L, Boyen F, Ducatelle R, et al. Effect of sedation on the intrapulmonary position of a bronchoalveolar lavage catheter in calves. Vet Rec. 2016;179:18. 10.1136/vr.103676 27114405

[18] Clinical and Laboratory Standards Institute. Performance standards for antimicrobial disk and dilution susceptibility tests for bacteria isolated from animals. Approved standard, 5th edition. CLSI document VET01–S3. Wayne (PA): The Institute; 2015.

[19] Clinical and Laboratory Standards Institute. Performance standards for antimicrobial disk and dilution susceptibility tests for bacteria isolated from animals. Approved standard, 28th edition. CLSI document VET08–S4. Wayne (PA): The Institute; 2018.

[20] Leinonen R, Akhtar R, Birney E, Bower L, Cerdeno-Tárraga A, Cheng Y, et al. The European Nucleotide Archive. Nucleic Acids Res. 2011;39(Database):D28–31. 10.1093/nar/gkq967 PMC3013801 20972220

[21] Wood DE, Salzberg SL. Kraken: ultrafast metagenomic sequence classification using exact alignments. Genome Biol. 2014;15:R46. 10.1186/gb-2014-15-3-r46 24580807 PMC4053813

[22] O’Leary NA, Wright MW, Brister JR, Ciufo S, Haddad D, McVeigh R, et al. Reference sequence (RefSeq) database at NCBI: current status, taxonomic expansion, and functional annotation. Nucleic Acids Res. 2016;44(D1):D733–45. 10.1093/nar/gkv1189 26553804 PMC4702849

[23] Bogaerts B, Winand R, Fu Q, Van Braekel J, Ceyssens PJ, Mattheus W, et al. Validation of a bioinformatics workflow for routine analysis of whole-genome sequencing data and related challenges for pathogen typing in a European National Reference Center: *Neisseria meningitidis* as a proof-of-concept. Front Microbiol. 2019;10:362. 10.3389/fmicb.2019.00362 30894839 PMC6414443

[24] Zankari E, Hasman H, Cosentino S, Vestergaard M, Rasmussen S, Lund O, et al. Identification of acquired antimicrobial resistance genes. J Antimicrob Chemother. 2012;67:2640–4. 10.1093/jac/dks261 22782487 PMC3468078

[25] Rice P, Longden I, Bleasby A. EMBOSS: the European Molecular Biology Open Software Suite. Trends Genet. 2000;16:276–7. 10.1016/S0168-9525(00)02024-2 10827456

[26] Arredondo-Alonso S, Rogers MRC, Braat JC, Verschuuren TD, Top J, Corander J, et al. mlplasmids: a user-friendly tool to predict plasmid- and chromosome-derived sequences for single species. Microb Genom. 2018;4:4. 10.1099/mgen.0.000224 30383524 PMC6321875

[27] Siguier P, Perochon J, Lestrade L, Mahillon J, Chandler M. ISfinder: the reference centre for bacterial insertion sequences. Nucleic Acids Res. 2006;34:D32–6. 10.1093/nar/gkj014 16381877 PMC1347377

[28] Liu M, Li X, Xie Y, Bi D, Sun J, Li J, et al. ICEberg 2.0: an updated database of bacterial integrative and conjugative elements. Nucleic Acids Res. 2019;47(D1):D660–5. 10.1093/nar/gky1123 30407568 PMC6323972

[29] Jolley KA, Maiden MCJ. BIGSdb: Scalable analysis of bacterial genome variation at the population level. BMC Bioinformatics. 2010;11:595. 10.1186/1471-2105-11-595 21143983 PMC3004885

[30] Jolley KA, Bray JE, Maiden MCJ. A RESTful application programming interface for the PubMLST molecular typing and genome databases. Database (Oxford). 2017;2017:bax060. 10.1093/database/bax060 29220452 PMC5550937

[31] Camacho C, Coulouris G, Avagyan V, Ma N, Papadopoulos J, Bealer K, et al. BLAST+: architecture and applications. BMC Bioinformatics. 2009;10:421. 10.1186/1471-2105-10-421 20003500 PMC2803857

[32] Kaas RS, Leekitcharoenphon P, Aarestrup FM, Lund O. Solving the problem of comparing whole bacterial genomes across different sequencing platforms. PLoS One. 2014;9:e104984. 10.1371/journal.pone.0104984 25110940 PMC4128722

[33] Letunic I, Bork P. Interactive Tree Of Life (iTOL) v4: recent updates and new developments. Nucleic Acids Res. 2019;47(W1):W256–9. 10.1093/nar/gkz239 30931475 PMC6602468

[34] Johnson TJ, Fernandez-Alarcon C, Bojesen AM, Nolan LK, Trampel DW, Seemann T. Complete genome sequence of *Gallibacterium anatis* strain UMN179, isolated from a laying hen with peritonitis. J Bacteriol. 2011;193:3676–7. 10.1128/JB.05177-11 21602325 PMC3133324

[35] Piddock LJ. Mechanisms of fluoroquinolone resistance: an update 1994-1998. Drugs. 1999;58(Suppl 2):11–8. 10.2165/00003495-199958002-00003 10553699

[36] Alispahic M, Christensen H, Hess C, Razzazi-Fazeli E, Bisgaard M, Hess M. Identification of *Gallibacterium species* by matrix-assisted laser desorption/ionization time-of-flight mass spectrometry evaluated by multilocus sequence analysis. Int J Med Microbiol. 2011;301:513–22. 10.1016/j.ijmm.2011.03.001 21596619

[37] El-Adawy H, Bocklisch H, Neubauer H, Hafez HM, Hotzel H. Identification, differentiation and antibiotic susceptibility of *Gallibacterium* isolates from diseased poultry. Ir Vet J. 2018;71:5. 10.1186/s13620-018-0116-2 29441195 PMC5799919

[38] Alispahic M, Christensen H, Hess C, Razzazi-Fazeli E, Bisgaard M, Hess M. MALDI-TOF mass spectrometry confirms clonal lineages of *Gallibacterium anatis* between chicken flocks. Vet Microbiol. 2012;160:269–73. 10.1016/j.vetmic.2012.05.032 22728126

[39] Bojesen AM, Torpdahl M, Christensen H, Olsen JE, Bisgaard M. Genetic diversity of *Gallibacterium anatis* isolates from different chicken flocks. J Clin Microbiol. 2003;41:2737–40. 10.1128/JCM.41.6.2737-2740.2003 12791918 PMC156494

[40] Magiorakos AP, Srinivasan A, Carey RB, Carmeli Y, Falagas ME, Giske CG, et al. Multidrug-resistant, extensively drug-resistant and pandrug-resistant bacteria: an international expert proposal for interim standard definitions for acquired resistance. Clin Microbiol Infect. 2012;18:268–81. 10.1111/j.1469-0691.2011.03570.x 21793988

[41] Portis E, Lindeman C, Johansen L, Stoltman G. A ten-year (2000-2009) study of antimicrobial susceptibility of bacteria that cause bovine respiratory disease complex—*Mannheimia haemolytica, Pasteurella multocida, and Histophilus somni*—in the United States and Canada. J Vet Diagn Invest. 2012;24:932–44. 10.1177/1040638712457559 22914822

[42] de Jong A, Thomas V, Simjee S, Moyaert H, El Garch F, Maher K, et al. Antimicrobial susceptibility monitoring of respiratory tract pathogens isolated from diseased cattle and pigs across Europe: the VetPath study. Vet Microbiol. 2014;172:202–15. 10.1016/j.vetmic.2014.04.008 24837878

[43] Michael GB, Bossé JT, Schwarz S. Antimicrobial resistance in *Pasteurellaceae* of veterinary origin. Microbiol Spectr. 2018;6:1–33. 10.1128/microbiolspec.ARBA-0022-2017 29916344 PMC11633590

[44] Cherkaoui A, Gaïa N, Baud D, Leo S, Fischer A, Ruppe E, et al. Molecular characterization of fluoroquinolones, macrolides, and imipenem resistance in *Haemophilus influenzae*: analysis of the mutations in QRDRs and assessment of the extent of the AcrAB-TolC-mediated resistance. Eur J Clin Microbiol Infect Dis. 2018;37:2201–10. 10.1007/s10096-018-3362-z 30145620

[45] Michael GB, Kadlec K, Sweeney MT, Brzuszkiewicz E, Liesegang H, Daniel R, et al. ICEPmu1, an integrative conjugative element (ICE) of *Pasteurella multocida*: analysis of the regions that comprise 12 antimicrobial resistance genes. J Antimicrob Chemother. 2012;67:84–90. 10.1093/jac/dkr406 22001175

[46] Eidam C, Poehlein A, Leimbach A, Michael GB, Kadlec K, Liesegang H, et al. Analysis and comparative genomics of ICEMh1, a novel integrative and conjugative element (ICE) of *Mannheimia haemolytica.* J Antimicrob Chemother. 2015;70:93–7. 10.1093/jac/dku361 25239467

[47] Johnson TJ, Danzeisen JL, Trampel D, Nolan LK, Seemann T, Bager RJ, et al. Genome analysis and phylogenetic relatedness of *Gallibacterium anatis* strains from poultry. PLoS One. 2013;8:e54844. 10.1371/journal.pone.0054844 23359626 PMC3554606

[48] Kristensen BM, Sinha S, Boyce JD, Bojesen AM, Mell JC, Redfield RJ. Natural transformation of *Gallibacterium anatis.* Appl Environ Microbiol. 2012;78:4914–22. 10.1128/AEM.00412-12 22582057 PMC3416388

