# Supplementary material for: Isolation of Drug-Resistant Gallibacterium anatis from Calves with Unresponsive Bronchopneumonia, Belgium
Source: Emerg Infect Dis. 2020 Apr;26(4):721–30. doi: 10.3201/eid2604.190962 (PMC7101127; doi:10.3201/eid2604.190962)
Supplement: Appendix — Additional information about isolation of drug-resistant Gallibacterium anatis from calves with unresponsive bronchopneumonia, Belgium. [file 19-0962-Techapp-s1.pdf]

Article DOI: https://doi.org/10.3201/eid2604.190962

Isolation of Drug-Resistant *Gallibacterium anatis* from Calves with Unresponsive Bronchopneumonia, Belgium

Appendix

Materials and Methods

Whole-Genome Sequencing

Trimmomatic 0.36 (*1*) was first used to trim raw reads setting the following options: "ILLUMINACLIP: NexteraPE-PE.fa:2:30:10", "LEADING:10", "TRAILING:10", "SLIDINGWINDOW:4:20", and "MINLEN:40". Afterwards, trimmed reads were de novo assembled using SPAdes 3.10.0 (*2*) setting the following options: "-careful", and "–cov-cutoff off". Orphaned reads resulting from trimming (i.e., reads where only one read of the pair survived) were also provided to the assembler as unpaired reads. Assembly statistics such as genome size, N50 (the length at which contigs of equal or longer length contain at least 50% of the assembled sequence), and number of contigs >1000 bases were calculated with QUAST 4.4 (*3*) using default settings, and are presented in Appendix Table 2.

Virulence Genotyping

Genotypic virulence gene detection was performed as described for antimicrobial resistance genotyping (see main article), but using the VirulenceFactor (*4*) full database (database accessed 04/03/2019). Results are presented in Appendix Table 4. Only one virulence gene, namely FimC (http://www.mgc.ac.cn/cgi-bin/VFs/gene.cgi?GeneID = VFG004079) coding for a type-1 fimbrial protein, was detected in some isolates.

SNP-Based Phylogenetic Analysis

Phylogenetic analysis was done using an in-house copy of the CSI phylogeny pipeline as follows: Trimmed reads (see "Whole genome sequencing") were used for read mapping against the NCBI RefSeq Genome entry for *G. anatis* (accession number NC_015460) for every sample with Bowtie2 2.3.0 (*5*) setting the following options: "–end-to-end", "–phred33", and "–

sensitive”. The “mpileup” program of Samtools 1.3.1 (*6*) was then used to create pileups setting the following options: “–count-orphans”, and “–VCF”, after which the “call” program of Bcftools 1.9 (*7*) was used to call SNPs setting the following options: “-O’ z”, “–consensus-caller”, “–variants-only”, “–ploidy 1”, and “–skip-variants indels”. The “filter” program of Bcftools was used to apply several quality filters to called SNPs by setting the following options: having a SNP depth of at least 10x with at least one forward and reverse read covering the position (“–exclude “DP<10 || DP4[0]+DP4[2]<1 || DP4[1]+DP4[3]<1”); having a SNP quality of at least 25 (“–exclude QUAL<25”); and having a mapping quality of at least 30 (“–exclude MQ<30”). Custom in-house scripts were used to apply two additional filters: keeping only one randomly selected SNP if two or more SNPs were located within the same window of 10 bases; and having a minimal Z-score and Y-multiplier of 1.96 and 10 (*8*), respectively.

cgMLST-based Phylogenetic Analysis

All 27 publically available genomes for *G. anatis* in the NCBI genome database were downloaded on 17/09/2019. An overview of the accession numbers for these samples is provided in Appendix Table 5. These genomes together with the assemblies of all Belgian isolates were used to construct a de novo cgMLST scheme with chewBBACA v2.0.17.2 (*9*). A prodigal training file was created using Prodigal v2.6.3 (*10*) using the NCBI RefSeq Genome entry for *G. anatis* (accession number NC_015460) as input and setting the “-p” parameter to “single”. A draft cgMLST scheme was then created using the chewBBACA “CreateScheme” function and setting the “–ptf” option to the aforementioned training file and providing all genomes as input. The “AlleleCall” function was used to perform allele calling for all loci in the draft scheme. Afterwards, the “RemoveGenes” function was used to remove paralogs and duplicate loci. Allele calling on the resulting scheme was done as described by Bogaerts et al., 2019 (*11*). A phylogeny based on the allele call matrix was created using GrapeTree 2.0 (*12*) setting the “method” option to “MSTreeV2”, and afterwards visualized within the GrapeTree interface. Host information for the NCBI genomes was retrieved from the “FEATURES” sections in their corresponding GenBank files.

References

1. Bolger AM, Lohse M, Usadel B. Trimmomatic: a flexible trimmer for Illumina sequence data. Bioinformatics. 2014;30:2114–20. PubMed https://doi.org/10.1093/bioinformatics/btu170

2. Bankevich A, Nurk S, Antipov D, Gurevich AA, Dvorkin M, Kulikov AS, et al. SPAdes: a new genome assembly algorithm and its applications to single-cell sequencing. J Comput Biol. 2012;19:455–77. PubMed https://doi.org/10.1089/cmb.2012.0021

3. Gurevich A, Saveliev V, Vyahhi N, Tesler G. QUAST: quality assessment tool for genome assemblies. Bioinformatics. 2013;29:1072–5. PubMed https://doi.org/10.1093/bioinformatics/btt086

4. Liu B, Zheng D, Jin Q, Chen L, Yang J. VFDB 2019: a comparative pathogenomic platform with an interactive web interface. Nucleic Acids Res. 2019;47(D1):D687–92. PubMed https://doi.org/10.1093/nar/gky1080

5. Langmead B, Salzberg SL. Fast gapped-read alignment with Bowtie 2. Nat Methods. 2012;9:357–9. PubMed https://doi.org/10.1038/nmeth.1923

6. Li H, Handsaker B, Wysoker A, Fennell T, Ruan J, Homer N, et al.; 1000 Genome Project Data Processing Subgroup. The Sequence Alignment/Map format and SAMtools. Bioinformatics. 2009;25:2078–9. PubMed https://doi.org/10.1093/bioinformatics/btp352

7. Li H. A statistical framework for SNP calling, mutation discovery, association mapping and population genetical parameter estimation from sequencing data. Bioinformatics. 2011;27:2987–93. PubMed https://doi.org/10.1093/bioinformatics/btr509

8. Kaas RS, Leekitcharoenphon P, Aarestrup FM, Lund O. Solving the problem of comparing whole bacterial genomes across different sequencing platforms. PLoS One. 2014;9:e104984. PubMed https://doi.org/10.1371/journal.pone.0104984

9. Silva M, Machado MP, Silva DN, Rossi M, Moran-Gilad J, Santos S, et al. chewBBACA: A complete suite for gene-by-gene schema creation and strain identification. Microb Genom. 2018;4:4. PubMed https://doi.org/10.1099/mgen.0.000166

10. Hyatt D, Chen GL, Locascio PF, Land ML, Larimer FW, Hauser LJ. Prodigal: prokaryotic gene recognition and translation initiation site identification. BMC Bioinformatics. 2010;11:119. PubMed https://doi.org/10.1186/1471-2105-11-119

11. Bogaerts B, Winand R, Fu Q, Van Braekel J, Ceyssens PJ, Mattheus W, et al. Validation of a bioinformatics workflow for routine analysis of whole-genome sequencing data and related challenges for pathogen typing in a European National Reference Center: *Neisseria meningitidis* as a proof-of-concept. Front Microbiol. 2019;10:362. PubMed https://doi.org/10.3389/fmicb.2019.00362

12. Zhou Z, Alikhan NF, Sergeant MJ, Luhmann N, Vaz C, Francisco AP, et al. GrapeTree: visualization of core genomic relationships among 100,000 bacterial pathogens. Genome Res. 2018;28:1395–404. PubMed https://doi.org/10.1101/gr.232397.117

Appendix Table 1. Genotypic resistance determinants and their corresponding MIC values for 16 antimicrobials commonly used to treat infectious bronchopneumonia of all investigated bovine *G. anatis* isolates

Isolate name	Antimicrobial agent	MIC (µg/mL)	Resistance gene/mutation
GB2	Penicillin	2	/
	Ampicillin	2	*erm*(B)
	Ceftiofur	≤0.03	*sul*2
	Amoxi/clav	≤0.12/0.06	*tet*(M)
	Tylosin	128	*cat*A1
	Tilmicosin	64	*cat*A3
	Tulathromycin	>128	*flo*R
	Trim/sulfa	16/304	*aad*A1
	Tetracycline	64	*aad*B
	Doxycycline	16	*aph*A1
	Florfenicol	32	*str*A
	Spectinomycin	128	*str*B
	Gentamicin	8	*gyr*A 83S→Y
	Kanamycin	>128	*gyr*A 87D→A
	Enrofloxacin	16	*par*C 80S→I
GB3	Penicillin	>128	*bla*CARB-8
	Ampicillin	>128	*bla*-TEM-2
	Ceftiofur	0.06	*erm*(B)
	Amoxi/clav	2/1	*sul*1
	Tylosin	128	*sul*2
	Tilmicosin	64	*tet*(B)
	Tulathromycin	128	*tet*(M)
	Trim/sulfa	2/38	*tet*(Y)
	Tetracycline	128	*flo*R
	Doxycycline	32	*aad*A1
	Florfenicol	32	*aad*B
	Spectinomycin	>128	*aph*A1
	Gentamicin	8	*str*A
	Kanamycin	>128	*str*B
	Enrofloxacin	16	*gyr*A 83S→Y
			*gyr*A 87D→A
			*par*C 80S→I
GB4	Penicillin	128	*bla*-TEM-2
	Ampicillin	>128	*erm*(B)
	Ceftiofur	0.06	*dfr*A1
	Amoxi/clav	2/1	*sul*2
	Tylosin	>128	
	Tilmicosin	128	*tet*(B)
	Tulathromycin	>128	*tet*M)
	Trim/sulfa	16/304	*cat*A1
	Tetracycline	128	*aac*(6’)-*aph*(2”)-1
	Doxycycline	16	*aad*A1
	Florfenicol	32	*aph*(3′)-III
	Spectinomycin	128	*str*A
	Gentamicin	32	*gyr*A 83S→Y
	Kanamycin	>128	*gyr*A 87D→A
	Enrofloxacin	32	*par*C 80S→I
GB5	Penicillin	0.5	/
	Ampicillin	1	*erm*(B)
	Ceftiofur	≤0.03	*dfr*A1
	Amoxi/clav	0.25/0.125	*sul*2
	Tylosin	>128	*tet*(B)
	Tilmicosin	128	*tet*(M)
	Tulathromycin	>128	*cat*A1
	Trim/sulfa	32/608	*flo*R
	Tetracycline	128	*aad*A1
	Doxycycline	16	*aad*B
	Florfenicol	4	*aph*A1

Isolate name	Antimicrobial agent	MIC (µg/mL)	Resistance gene/mutation
	Spectinomycin	128	*str*A
	Gentamicin	8	*gyr*A 83S→Y
	Kanamycin	>128	*gyr*A 87D→A
	Enrofloxacin	16	*par*C 80S→I
GB6	Penicillin	128	*bla*-CARB-8
	Ampicillin	>128	*bla*-TEM-2
	Ceftiofur	0.06	*erm*(B)
	Amoxi/clav	2/1	*dfrA*1
	Tylosin	>128	*sul*1
	Tilmicosin	64	*sul*2
	Tulathromycin	>128	*tet*(B)
	Trim/sulfa	32/608	*tet*(M)
	Tetracycline	128	*tet*(Y)
	Doxycycline	32	*flo*R
	Florfenicol	8	*aad*A1
	Spectinomycin	128	*aph*A1
	Gentamicin	8	*str*A
	Kanamycin	>128	*str*B
	Enrofloxacin	16	*gyr*A 83S→F
			*gyr*A 87D→G
			*par*C 80S→I
GB7	Penicillin	>128	*bla*-TEM-2
	Ampicillin	>128	*erm*(B)
	Ceftiofur	≤0.03	*su*l2
	Amoxi/clav	2/1	*tet*(B)
	Tylosin	128	*tet*(M)
	Tilmicosin	128	*cat*A1
	Tulathromycin	32	*cat*A3
	Trim/sulfa	32/608	*aad*A1
	Tetracycline	128	*aad*B
	Doxycycline	16	*aph*A1
	Florfenicol	1	*str*A
	Spectinomycin	>128	*str*B
	Gentamicin	16	*gyr*A 83S→F
	Kanamycin	>128	*gyr*A 87D→G
	Enrofloxacin	16	*par*C 80S→I
GB8	Penicillin	>128	*bla*-TEM-2
	Ampicillin	>128	*erm*(B)
	Ceftiofur	0.06	*mph*(E)
	Amoxi/clav	1/0.5	*mrs*(E)
	Tylosin	>128	*dfrA*1
	Tilmicosin	>128	*su*l2
	Tulathromycin	>128	*tet*(B)
	Trim/sulfa	>128/2432	*tet*(M)
	Tetracycline	>128	*cat*A1
	Doxycycline	32	*cat*A3
	Florfenicol	1	*aad*A23
	Spectinomycin	>128	*aad*B
	Gentamicin	>128	*aph*A1
	Kanamycin	>128	*str*A
	Enrofloxacin	8	*gyr*A 83S→F
			*gyr*A 87D→A
			*par*C 80S→I
GB9	Penicillin	128	*bla*-TEM-2
	Ampicillin	>128	*erm*(B)
	Ceftiofur	≤0.03	*dfr*A1
	Amoxi/clav	2/1	*sul*2
	Tylosin	>128	*tet*(B)
	Tilmicosin	128	*tet*(M)
	Tulathromycin	>128	*cat*A1
	Trim/sulfa	16/304	*aac*(6′)-*aph*(2”)-1
	Tetracycline	>128	*aad*A1
	Doxycycline	16	*aph*(3′)-III
	Florfenicol	1	*str*A
	Spectinomycin	128	*gyr*A 83S→Y
	Gentamicin	>128	*gyr*A 87D→A

Isolate name	Antimicrobial agent	MIC (µg/mL)	Resistance gene/mutation
	Kanamycin	>128	*par*C 80S→I
	Enrofloxacin	32	
GB10	Penicillin	>128	/
	Ampicillin	>128	*erm*(B)
	Ceftiofur	≤0.03	*sul*2
	Amoxi/clav	3/1.5	*tet*(B)
	Tylosin	128	*tet*(M)
	Tilmicosin	16	*cat*A1
	Tulathromycin	4	*flo*R
	Trim/sulfa	32/608	*aad*A1
	Tetracycline	128	*aad*B
	Doxycycline	8	*aph*A1
	Florfenicol	8	*str*A
	Spectinomycin	>128	
	Gentamicin	0.5	*qnr*D1
	Kanamycin	>128	*gyr*A 83S→Y
	Enrofloxacin	16	*gyr*A 87D→A
			*par*C 80S→I
GB11	Penicillin	128	*bla*-TEM-2
	Ampicillin	>128	*erm*(B)
	Ceftiofur	0.25	*dfr*A1
	Amoxi/clav	0.5/0.25	*sul*2
	Tylosin	>128	*tet*(B)
	Tilmicosin	>128	tet(M)
	Tulathromycin	>128	
	Trim/sulfa	32/608	*cat*A1
	Tetracycline	128	
	Doxycycline	16	*aac*(6′)-*aph*(2”)
	Florfenicol	1	*aad*A1
	Spectinomycin	>128	*aph*(3′)-III
	Gentamicin	32	*str*A
	Kanamycin	>128	*gyr*A 83S→Y
	Enrofloxacin	32	*gyr*A 87D→A
			*par*C 80S→I

Appendix Table 2. Overview of WGS summary statistics expressed as number of raw paired-end reads, genome assembly length, N50, and number of contigs >1,000 bases

Isolate name	No. paired-end reads	Genome assembly length	N50	No. contigs >1,000 bases
GB2	428,631	2,549,575	80,564	62
GB3	375,338	2,440,244	99,746	57
GB4	332,655	2,398,744	110,501	53
GB5	276,532	2,507,524	89,941	65
GB6	382,662	2,427,176	89,568	59
GB7	344,368	2,524,470	129,663	55
GB8	326,968	2,466,991	68,465	74
GB9	452,788	2,597,989	122,205	73
GB10	369,907	2,499,083	157,729	48
GB11	380,273	2,352,964	131,690	44

Appendix Table 3. Mapping rates and number of SNPs after filtering for all isolates using either *G. anatis* UMN179 or GB8 as reference

Isolate name	Reference = *G. anatis* UMN179		Reference = GB8	
	Mapping rate, %	No. SNPs after filtering	Mapping rate, %	No. SNPs after filtering
GB2	71.26	15,189	72.35	10,855
GB3	77.43	14,597	84.21	8,978
GB4	76.71	14,767	82.14	9,173
GB5	77.73	14,583	78.33	10,688
GB6	78.28	14,593	84.84	8,979
GB7	78.15	15,162	76.61	11,137
GB8	80.22	14,795	95.4	1
GB9	74.36	14,814	79.41	9,216
GB10	71.36	15,234	73.15	10,941
GB11	78.54	14,967	85.01	9,303

Appendix Table 4. Overview of NCBI accession numbers for all *G. anatis* isolates used for constructing a cgMLST scheme and resulting topology

Name	Accession number (NCBI assembly)
GCF_000209675	GCF_000209675.1_ASM20967v1
GCF_000379785	GCF_000379785.1_ASM37978v1
GCF_000464615	GCF_000464615.2_Ga_12656_12_1.0
GCF_000771775	GCF_000771775.1_ASM77177v1
GCF_000771785	GCF_000771785.1_ASM77178v1
GCF_000771795	GCF_000771795.1_ASM77179v1
GCF_000771805	GCF_000771805.1_ASM77180v1
GCF_000771855	GCF_000771855.1_ASM77185v1
GCF_000771915	GCF_000771915.1_ASM77191v1
GCF_000771935	GCF_000771935.1_ASM77193v1
GCF_000771955	GCF_000771955.1_ASM77195v1
GCF_000771975	GCF_000771975.1_ASM77197v1
GCF_000772265	GCF_000772265.1_ASM77226v1
GCF_000772275	GCF_000772275.1_ASM77227v1
GCF_000772285	GCF_000772285.1_ASM77228v1
GCF_000772295	GCF_000772295.1_ASM77229v1
GCF_000772345	GCF_000772345.1_ASM77234v1
GCF_000772365	GCF_000772365.1_ASM77236v1
GCF_000772385	GCF_000772385.1_ASM77238v1
GCF_000772395	GCF_000772395.1_ASM77239v1
GCF_000772425	GCF_000772425.1_ASM77242v1
GCF_000772445	GCF_000772445.1_ASM77244v1
GCF_001678465	GCF_001678465.1_Gal26
GCF_001678565	GCF_001678565.1_Gal27
GCF_002263255	GCF_002263255.1_ASM226325v1
GCF_002263295	GCF_002263295.1_ASM226329v1
GCF_900450735	GCF_900450735.1_49950_E01

Appendix Table 5. Overview of all hits for the VirulenceFactor full database

Sample name	Locus detected	% Identity	HSP/Locus length	Contig	Position in contig
GB2	Fim (CVF003)	98.59	1347/2052	NODE_3_length_239488_cov_37.058585	162028..163374
GB3	Fim (CVF003)	100	1237/2052	NODE_13_length_55171_cov_37.453256	45256..46492
GB4	Fim (CVF003)	99.76	1240/2052	NODE_13_length_56472_cov_27.581205	1..1240
GB5	Fim (CVF003)	99.68	1253/2052	NODE_12_length_71610_cov_28.199852	1..1253
GB6	Fim (CVF003)	100	1237/2052	NODE_13_length_55173_cov_41.462704	8680..9916
GB9	Fim (CVF003)	99.92	1241/2052	NODE_25_length_22235_cov_39.928714	1..1241
GB11	Fim (CVF003)	98.81	1347/2052	NODE_9_length_76651_cov_35.885030	55233..56579

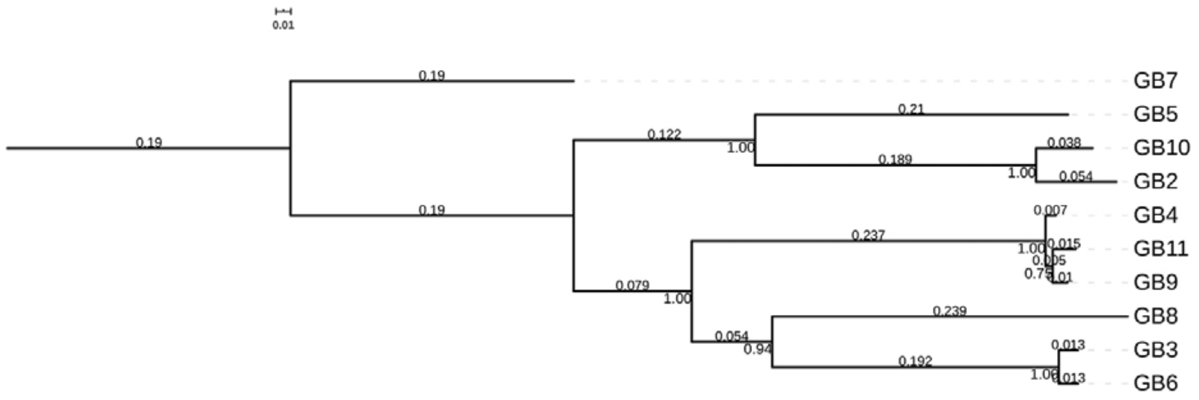

Appendix Figure. Phylogeny of *Gallibacterium anatis* isolates based on SNP genotyping when using *G. anatis* UMN179 as a reference. Node labels indicate bootstrap support values (expressed as decimals). Branch lengths and the scale bar are expressed as average substitutions per site.
